# Does increasing public expenditure on sports promote regional sustainable development: Evidence from China

**DOI:** 10.3389/fpubh.2022.976188

**Published:** 2022-09-21

**Authors:** Dingqing Wang, Enqi Zhang, Peng Qiu, Xiaoyu Hong

**Affiliations:** ^1^School of Economics, Jilin University, Changchun, China; ^2^Krieger School of Arts and Sciences, Johns Hopkins University, Washington, DC, United States; ^3^Physical Education College, Jilin University, Changchun, China; ^4^Business School, Nanjing University, Nanjing, China

**Keywords:** regional sustainable development, public expenditure on sports, residents' consumption, threshold effect, sports economic

## Abstract

In the post-COVID era, how to improve the level of regional sustainable development has attracted much attention. And the vigorous development of the sports economy may be closely related to the regional sustainable development. This paper explores the impact and mechanism analysis of government sports public expenditure on regional sustainable development from the perspective of sports economic development. The study found that China's sustainable development presents obvious ladder-like characteristics and highlights the regional imbalance and inadequacy of regional green and coordinated development. And the government's increase in public expenditure on sports can significantly promote regional sustainable development and improve the level of regional green and coordinated development. With the continuous improvement of the regional economic development, the effect of sports public expenditure continues to increase. It can be seen from this that implementing the strategy of strengthening the country through sports under the government's guidance is an essential guarantee for the public health and quality of life and the sustainable development of the economy and society. Additionally, the development level of market finance is also an important driving factor for the government's public expenditure on sports to improve the level of sustainable development in the region. From the mechanism analysis, the government activates the local residents' consumption level by increasing the public expenditure on sports, thus promoting regional sustainable development.

## Introduction

The civilizational progress of human society is essentially the history of the evolution of the relationship between man and nature. In addition, how to deal with the relationship between man and society, man and nature, and realize the coordinated development and win–win symbiosis of the economy and society has become a challenge faced by all humankind. Sustainable development is a multidimensional concept that emphasizes the integration of the economy, society and environment within a region and the realization of dynamic balance (1, 2). According to the interpretation of sustainable development made by the International Union of Ecology (INTECOL), the United Nations Environment Programme (UNEP), and other departments, sustainable development mainly emphasizes the importance of ecological civilization construction in the natural environment, economy, society, science, and technology (3, 4), which shows the importance of green and coordinated development in sustainable development. The United Nations' 2030 Sustainable Development Goals, a series of conventions on global climate change, and countries' actions to eradicate poverty and positive responses to green development are all deepening the concept of sustainable development. Only by steadily advancing the transformation of the economic development mode and building a harmonious and symbiotic development situation can we effectively achieve sustainable economic and social development.

Regarding the current situation of China's development, on the one hand, the Chinese government has effectively completed the historical task of poverty alleviation of residents, but there are still apparent challenges of unbalanced and insufficient regional development (5). On the other hand, in the process of economic development, China's carbon emissions have rapidly increased, and it has surpassed the United States to become the largest carbon emitter. Its environmental carrying capacity has been severely tested (6). Obviously, the key to improving the quality of regional development is to solve the problems of unbalanced regional development and low quality and high consumption in the development process. Sustainable development is a new development concept from extensive growth to intensive development. It closely links economic development with green ecology, balance and coordination and has become an essential guarantee for the realization of high-quality development led by new development concepts. At the same time, as the socialist market economy with Chinese characteristics has entered a new stage of development, residents are not only satisfied with basic material needs but also pay increasing attention to the pursuit of beauty at the cultural and spiritual level, and the sports economy has emerged as the times require. Chinese leaders attach great importance to sports development from the strategic level of national prosperity and national rejuvenation. They put forward a series of new concepts, new ideas, and new strategies around accelerating the construction of a strong sports country, promoting the reform and innovation of sports, and implementing the national strategy for national fitness. The sports industry, represented by competitive sports, sports venue management, sports goods trading, sports insurance industry, sports tourism industry, sports advertising industry, and so on, plays an essential role in promoting the improvement of residents' quality of life, the optimization of industrial structure and the improvement of the national economy (7). The vigorous development of the sports economy has a profound impact on the quality of China's economic and social development, and the cause of sports and health is of great practical significance to green, coordinated, and sustainable development. As the helm of the region's sustainable development and the guide of the development of sports, can an effective government promote the development of sports by exercising public functions and then affecting the level of sustainable development in the region? What factors are likely to affect the effect of government action? What is its mechanism of action? How can the strategy of sports power be implemented in the post-COVID era? We will discuss the above issues.

Improving the level of regional sustainable development has become a hot issue in academic circles. Some scholars have constructed sustainable development indicators and conducted comparative analyses. Lu et al. (8) constructed a regional sustainable development index based on a driver-pressure-state-impact-response model, which contributed to the evaluation and analysis of sustainable development. Ding et al. (9) constructed urban sustainable development indicators from three aspects, society, economy, and environment, and put forward suggestions for China's comprehensive, coordinated and sustainable development based on analysis and evaluation. Existing scholars' research on regional sustainable development capacity mainly focuses on the equity of social development and the sustainability of the resource and environmental carrying capacity (10, 11). Some scholars have explored the influencing factors of regional green coordinated development from the aspects of energy consumption (12), regional carbon emissions (13, 14), urbanization and industrial structure upgrading (15), technological innovation and governance institution quality (16), the degree of marketization and legal environment (17), and the regional spatial structure (18). Gu et al. (19) further analyzed the impact path of entrepreneurship level on regional sustainable development. The study found a strong link between entrepreneurship and regional sustainable development. It also found that the business environment and foreign direct investment not only have a direct impact on the triple bottom line of sustainable development but also have a significant moderating effect on the impact of entrepreneurship on regional sustainable development. Dabbous and Tarhini (20) explored the potential impact of the sharing economy on regional sustainable development. The author used Google Internet big data to further verify that the sharing economy can stimulate regional sustainable development. Sports economics is a young subject area (21), and the sports industry is an industrial sector with strong public attributes. Existing research on the spillover of the economic effects of sports is not common. On the one hand, the existing research mainly focuses on the development analysis of the sports industry and the sports cause itself. Moreover, its research methods mainly include literature analysis (22, 23), interviews and questionnaires (24, 25), and simulation analysis (7, 26). On the other hand, in promoting regional economic development, the government plays an essential role through basic public expenditure (27). However, few studies have explored the impact of government public expenditure on regional sustainable development from the perspective of the sports economy (28) explored the relationship between the government's spending on sports and residents' participation in physical activity by combining individual survey data with state-level data. The study found that the government's continuous expenditure on sports facilities has a significant positive relationship with residents' participation in physical exercise and effectively promotes the improvement of public health. Kim (29) explored the impact of sports venues and sports-related activities on regional economic development and performed a quantitative analysis from the perspective of sports business and the economic spillover of the sports industry.

There is currently a lack of research on the impact of government behavior on regional sustainable development in the process of sports development. We extend the literature mentioned above on the impact of public expenditure on sports on regional sustainable development and verify the vital impact of government behavior on the development of sports public utility on regional sustainable development from a theoretical and empirical perspective.

Our research found that the government will guide the orderly advancement of sports by increasing public expenditure on sports. It could promote regional sustainable development and the improvement of greenness and coordination. Specifically, our marginal contribution is mainly reflected in the following aspects. First, in measuring the sustainable development level, this paper uses green development and coordinated development as the standards to construct a regional sustainable development index, which more representatively highlights the greening and coordination of sustainable development. Second, different from questionnaires, simulation analysis, and literature induction analysis, we use the relevant data on sports public fiscal expenditures of provincial government departments in China. Then, we take the level of economic development as the threshold variable. From the perspective of public fiscal support, the dynamic effect of the development of sports public utilities on the sustainable development of the region is verified. Moreover, we found that with the improvement of the level of regional economic development, the positive promotion of the development of sports public utilities has been continuously strengthened. Third, from the perspective of the demand effect, we verify the transmission mechanism by which the government promotes the regional consumption level through sports expenditure to promote sustainable development and explore the impact of the market financial development level on the effect of government expenditure. We found a positive mediating effect on regional residents' consumption level and a moderating effect on market financial development level.

## Theoretical analysis and hypothesis development

In the process of sustainable development, sports undertakings and the sports industry gradually show their own economic advantages (7). Asian countries represented by China have gradually become essential players in the international sports business (30). It is of great practical significance to explore the impact of sports on regional development. The Chinese government clearly stated in the mid-term planning document for the development of sports, “Actively promote the coordinated development of sports and the economy and society, and continuously enhance the systematicness and synergy of various sports work. Promote the coordinated development of sports undertakings and the sports industry and the comprehensive development of mass sports and competitive sports. Promote the balanced development of urban and rural sports and the coordinated development of regional sports.” As a guide for the development of sports and a promoter of sustainable development in the region, the Chinese government will guide and support the development of sports through financial expenditures to improve the level of sustainable development in the region.

From the perspective of the supply side, increasing the financial expenditure on sports can improve the coordination level of regional economic and social development. On the one hand, the sports convergence industry is emerging, and the orderly development of sports can promote the vigorous development of related service industries, product processing industries, and some advanced science and technology (31), thereby effectively enhancing the regional industrial development level of coordination. On the other hand, under the background of an obvious imbalance in the primary distribution, the government realizes wealth redistribution through fiscal transfer payments (32), enabling the balanced allocation of sports public resources among regions and promoting coordinated regional development. From the demand side, the government, through fiscal support to hold large-scale sports events, guides the public to change their lifestyles, attaches great importance to cultivating residents' sports and health habits, and advocates a low-carbon life, thereby promoting the green development of the region. “Athlete-centered, sustainable development, and frugal hosting” is the 2022 Beijing Winter Olympics bidding concept. Among them, the concept of “sustainable development” emphasizes “the development that meets the needs of contemporary people without compromising their future needs and gives full play to the role of the Olympic Movement in promoting and regulating the economy, society, and the natural environment,” which aims to integrate the concept of green development throughout our preparations. The concept of green development is closely related to residents' lives through the development of sports undertakings, and the sports economy is deeply integrated into residents' lives, gradually changing residents' lifestyles, promoting the development of an ecological society, and forming a virtuous circle of green ecological environment and economic and social development. Therefore, we suggest the following:

*Hypothesis 1:* Increasing the government's public spending on sports can improve the level of sustainable development in the region.

According to Petty-Clark's law (33), with the continuous development of the economy and society and the continuous improvement of per capita GDP, the regional economic structure will show a transition from the dominance of the primary industry to the dominance of the secondary and tertiary industries. Specifically, as the income level of residents increases, the status of the agricultural sector in the national economy gradually declines, and the secondary industry will gradually occupy a dominant position in the national economy due to its technological progress and economies of scale. As the income level of residents further increases, the income elasticity of demand for industrial products will gradually decrease; the income elasticity of demand for services, leisure products and services will increase; and labor will gradually be concentrated in such industries. The sports industry is a service and leisure industry. Under the effect of demand, the investment scale and structure of the sports industry will change accordingly. Driven by government fiscal support and investment, the sports industry is becoming increasingly prosperous, not only becoming a new economic growth point but also promoting the transformation and upgrading of the industrial structure. With the continuous improvement of residents' income level, residents' pursuit of physical and mental health and quality of life will be stronger, and investment in the sports industry can largely meet people's needs for health and leisure.

From the perspective of historical economic and social development experience, the more developed the economy is, the higher the status of the sports industry in the national economy (34). In Japan, the United Kingdom, Germany, France and other countries, the sports industry accounts for approximately 1–3% of GDP, and Switzerland accounts for 3.47%, which is the highest (35). Obviously, the sports economy has a substantial potential market value that has not yet been activated, making it the most dynamic and promising industry in the 21st century. From the perspective of economic structure changes, with the continuous improvement of the level of economic and social development, the proportion of the service industry in the economic structure is increasing day by day, and it has become a primary industrial sector with a strategic position in modern society (36). As an essential part of the service industry, the sports industry plays an important role in promoting the optimization of the industrial structure and promoting the sustainable development of the region, and its role in improving the quality of regional development is also increasing. Therefore, we put forward the following hypothesis:

*Hypothesis 2:* Public expenditure on sports has a non-linear impact on regional sustainable development. With the improvement of the level of regional economic development, the effect of public expenditure on sports on the region's sustainable development has been continuously enhanced.

As far as the history of China's economic and social development is concerned, the relationship between the government and the market has gone from the “single government playing a role” in the planned economy period to the third plenary session of the 18th Central Committee officially proposing that “the market plays a decisive role in the allocation of resources and make the role of the government play better.” Then, in the Fifth Plenary Session of the 19th Central Committee of the Communist Party of China, it was clearly pointed out that “We will fully leverage the decisive role of the market in allocating resources and give better play to the role of government, to ensure better alignment between an efficient market and a well-functioning government.” In the process of promoting the coordinated development of regional green, both the government and market forces play an essential role (37). The government's public expenditure on sports impacts the sustainable development of the region (38), and its effect will also be affected by market forces (39). In the context of the policy guidelines for correctly Handling the relationship between the government and the market, it is necessary to fully consider the impact of market development on the marginal contribution of government fiscal support. In the vigorous development of the sports economy, the effect of local government sports public expenditure on the quality of regional development may be affected by the level of market financial development. Under the dual-track economic resource allocation model of government and market, with the improvement of the development level of market finance, whether the impact of changes in local government sports public expenditure on sustainable economic development needs to be further tested. In summary, this paper proposes the following assumptions:

*Hypothesis 3:* The development level of market finance has a positive moderating effect on the promotion effect of sports public expenditure on regional sustainable development.

The increase in the government's public expenditure on sports can improve residents' actual income level, reduce precautionary savings and activate consumption diversity motivation through the construction of a soft environment, guide the development of the sports and health industry, and improve the level of regional economic development. These effectively enhance the ability and willingness of individuals to consume, promote the formation of a sizeable domestic circulation pattern, and thus promote the sustainable development of the region. According to the life cycle theory, the current consumption of an economic person is determined by his entire lifetime income, and people tend to smooth their consumption expenditures according to their lifetime income. Some scholars use this hypothesis to explain the “mystery of Chinese savings” (40, 41), arguing that rational individuals generally have preventive savings behaviors. On the one hand, the government accelerates the construction of sports infrastructure, effectively promotes the development of sports tourism and sports events, and improves the level of regional economic development, which increases its actual income level throughout life. It increases the willingness and motivation to consume and improves the quality of regional supply through the demand effect, thereby driving the region's sustainable development. On the other hand, the increase in public expenditure on sports could promote the development of the sports and health industry, guide consumption diversification and the improvement of consumption quality, and promote the optimization of consumption structure. Furthermore, the share of consumption used in the field of sports and health has gradually increased, which could activate the potential domestic demand market and promote the sustainable development of the region. Therefore, we put forward the following hypothesis:

*Hypothesis 4:* The increase in public expenditure on sports can promote regional consumption levels, activate the internal circulation market, and promote regional sustainable development.

## Research design

### Regression model

To verify the impact of the degree of emphasis on sports on the level of sustainable development in the region, that is, *Hypothesis 1*, we set the basic regression model:


(1) $$\begin{array}{l} sus_{it}=\beta_{0}+\beta_{1}sportscl_{it}+\beta_{2}r\&d_{it}+\beta_{3}patent_{it}\\ \;+\beta_{4}fdi_{it}+\beta_{5}energy_{it}+\beta_{6}gdp_{it}+\beta_{7}upd_{it}+\varepsilon_{it} \end{array}$$

Among them, *sus* represents sustainable development, and *sportscl* represents the degree of importance that the region attaches to sports undertakings, which is measured by the stock of fiscal expenditures for sports. The *r&d* represents the region's emphasis on scientific and technological development, which is measured by the rate of change in R&D investment. The *patent* represents the regional innovation foundation and atmosphere, which is measured by the number of invention patents granted per 10,000 people. The *fdi* represents the level of opening to the outside world, measured as the actual amount of foreign direct investment per capita. The *energy* represents the importance of green development in the region and is measured by the energy consumption per unit of GDP. The *gdp* represents the rate of regional economic growth, measured as the rate of change in GDP. The *upd* represents the degree of population agglomeration in the region, measured by the urban population density. β is the regression coefficient, and ε is a random interference term.

To test hypothesis 3, we investigate whether the development level of market finance will affect the promotion effect of sports public expenditure on the high-quality development of the region. We introduce the interaction term *fin*×*sportscl* between the level of financial development and regional sustainable development as an explanatory variable and the level of financial development (*fin*) as a moderator variable. We construct the regression model of Equation (2) and Equation (3) to explore the moderating effect of the development level of market finance.


(2) $$\begin{array}{l} sus_{it}=\partial_{0}+\partial_{1}sportscl_{it}+\partial_{2}fin_{it}+\overset{8}{\underset{j=3}{\sum }}\partial_{j}control_{jit}+\varepsilon_{it} \end{array}$$

(3) $$\begin{array}{l} sus_{it}=\alpha_{0}+\alpha_{1}sportscl_{it}+\alpha_{2}fin_{it}\times sportscl_{it}\\ \;+\overset{8}{\underset{j=3}{\sum }}\alpha_{j}control_{jit}+\varepsilon_{it} \end{array}$$

To test whether public expenditure on sports will affect the region's high-quality development by stimulating the regional residents' consumption level, which is to test *Hypothesis 4*, we draw on the method of Baron and Kenny (42). We use the mediating effect model, adopt the stepwise regression analysis method, and construct the regression model of Equation (4) and Equation (5). Then, we combine the two equations with Equation (1) to explore the impact and mechanism of sports public expenditure on regional sustainable development. Among them, the *consump* is the consumption level of regional residents.


(4) $$\begin{array}{l} consump_{it}=\gamma_{0}+\gamma_{1}sportscl_{it}+\overset{7}{\underset{j=2}{\sum }}\gamma_{j}control_{jit}+\varepsilon_{it} \end{array}$$

(5) $$\begin{array}{l} sus_{it}=\phi_{0}+\phi_{1}sportscl_{it}+\phi_{\text{2}}consump_{it}+\overset{8}{\underset{j=3}{\sum }}\phi_{j}control_{jit}\\ \;+\varepsilon_{it} \end{array}$$

### Variable descriptions

#### The indicators of regional sustainable development

Based on summarizing economic and social-historical development experience, the Chinese government has formulated a high-quality development strategy guided by new development concepts, and sustainable development based on green and coordinated development has become an important guarantee for high-quality development. The Chinese government issued an important document to make an important conclusion: “High-quality development is the development that can meet the people's ever-growing needs for a better life, the development that embodies the new vision of development, and the development in which innovation has become the primary driving force, coordination has become an endogenous feature, green development has become common, opening up has become the only way, and sharing has become the fundamental goal.”^1^ Based on this, driven by the goal of building a new development pattern and promoting regional sustainable development, it closely follows the basic requirements of “greenness and coordination.” According to the principles of feasibility and simplicity in the construction of sustainable development evaluation indicators, the original intention is to select appropriate indicators to reflect more results. We build a sustainable development evaluation system from the perspective of green development and coordinated development. The specific indicators are shown in Table 1.

**Table 1:** Index of regional sustainable development level.

**First-level indicators**	**Second-level indicators**	**Third-level indicators**	**Notes**
The regional sustainable development (*sus*)	Coordination (Z_1_)	Level of coordinated development of regional industries (Z_11_)	Degree of rationalization of industrial structure Z${}_{11}^{a}$
		Level of coordinated urban–rural development (Z_12_):	Urban–rural income gap (Income of rural residents/Income of urban residents) Z_12_
	Greenness (Z_2_)	Basic environmental change degree (Z_21 − 24_)	PM_2.5_ population weighted value Z_21_; Unit energy consumption to create GDP value Z_22_; Industrial solid waste utilization rate Z_23;_ Urban wastewater utilization rate Z_24_
		Development of environmental protection technology (Z_25_)	Number of green invention patents granted (mainly including seven categories such as alternative energy, transportation, energy conservation, waste management, agriculture and forestry, administrative supervision and design, and nuclear power) Z_25_

^a^This paper uses theil index to measure the degree of rationalization of industrial structure, and the calculation formula is

$z_{i,t}=\sum_{m=1}^{3}y_{i,m,t}\cdot \ln\;\left(y_{i,m,t}/l_{i,m,t}\right),m=1,2,3$.

The y_i, m, t_ represents the proportion of the m industry in region i in the GDP in period t, and the li,m,t represents the proportion of the m industry in region i in the total employment from one person in period t. In addition, the smaller the value of z, the more reasonable the industrial structure.

We draw on the processing method of Ding et al. (9), use the entropy weight TOPSIS model, and calculate the sustainable development index of 30 provinces (cities, districts) from 2006 to 2017 through the objective weight weighting method. Among them, the number of regional green patent authorizations is classified and sorted according to the number of green invention patent authorizations of listed companies in the patent database of the State Intellectual Property Office. ^2^Among the three-level indicators, Z_11_ and Z_21_ are negative indicators, and Z_12_ and Z_22_-Z_25_ are positive indicators.

#### The level of public expenditure on sports

We use the relevant data on sports public expenditure in various regions in the *China Sports Statistical Yearbook* as a measure of the level of sports public expenditure. In the robustness test, we use the fixed investment in sports culture in the *China Fixed Assets Statistical Yearbook* instead. In the process of data processing, we use 2006 as the initial year to calculate the public expenditure of sports undertakings based on the perpetual inventory method and perform logarithmic processing.

#### Description of mediating variables, moderating variables, and control variables

In addition to the level of sustainable development and public fiscal expenditures for sports, the mediating variable in this paper is the household consumption level (*consump*), which is measured by the statistical data published in the National Bureau of Statistics database. The moderating variable was financial development level (*fin*), which is measured by the ratio of the regional financial industry output value to the regional GDP. The control variables involved mainly include the strength of R&D support (*r&d*), the innovation foundation and atmosphere (*patent*), the level of opening to the outside world (*fdi*), the level of energy consumption (energy), the rate of economic growth (*gdp*), and the degree of population agglomeration (*upd*). Descriptive statistics of the related variables are shown in Table 2.

**Table 2:** Descriptive statistics for the variables.

**VarName**	**Obs**	**Mean**	**Median**	**SD**	**Min**	**Max**
*sus*	360	0.459	0.454	0.093	0.237	0.745
*coordination*	360	0.511	0.510	0.165	0.000	0.877
*greeness*	360	0.442	0.443	0.088	0.249	0.790
*sportscl*	360	12.901	13.000	0.828	9.717	14.527
*sportscl1*	360	12.993	13.070	1.321	8.711	15.262
*consump*	360	1.152	0.984	0.669	0.380	4.048
*fin*	360	0.058	0.052	0.030	0.016	0.177
*gdp_per*	360	3.798	3.298	2.308	0.614	13.765
*gdp*	360	0.135	0.123	0.065	−0.040	0.298
*fdi*	360	0.108	0.069	0.133	0.001	0.851
*energy*	360	1.089	0.934	0.617	0.239	4.142
*r&d*	360	2.186	−0.196	7.706	−0.946	66.562
*patent*	360	1.110	0.405	2.239	0.036	21.230
*upd*	360	0.279	0.256	0.123	0.060	0.631

### Data sources

On the one hand, the reform of fiscal expenditure items was carried out after 2006, and we need to ensure the consistency of the statistical caliber of data when selecting data. On the other hand, the 11th Five-Year Plan for 2006 clearly points out that “we should put people first, change our development philosophy, innovate the development model, improve the quality of development, implement the five overall plans, and put economic and social development on the track of comprehensive, coordinated and sustainable development.” Economic and social development focus is tilted toward the harmonious coexistence of man and nature, emphasizing comprehensive, coordinated and sustainable development. Therefore, under the principle of data availability, we select the relevant data of 30 provincial-level regions (excluding Tibet, Hong Kong, Macao and Taiwan) from 2006 to 2017. The data mainly come from the *China Sports Statistical Yearbook, China Statistical Yearbook, China Fixed Asset Investment Yearbook*, the database of the National Bureau of Statistics, the patent database of the State Intellectual Property Office, and the statistical yearbooks of various provinces and regions.

## Regression analysis

### Evaluation and of regional sustainable development index

**Figure 1:**
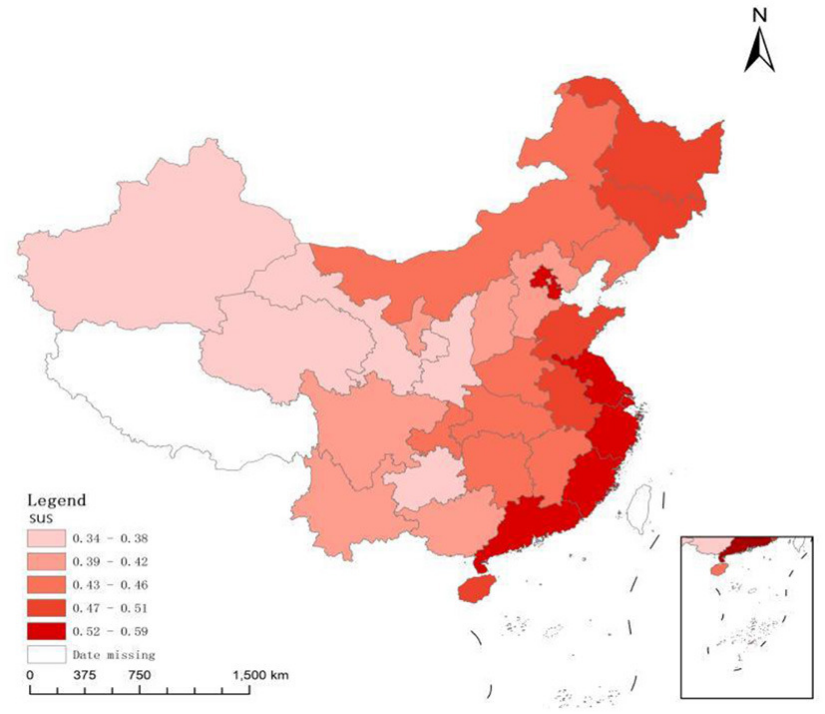
Average ranking of sustainable development of Chinese provinces^*a*^. ^*a*^The Drawing Review number is GS(2019)1822. And the source is http://bzdt.ch.mnr.gov.cn/download.html?searchText=GS(2019)1822.

Figure 1 is a distribution chart of the average value of the sustainable development index of Chinese provinces from 2006 to 2017. As far as provincial sustainable development is concerned, overall sustainable development of the eastern coastal areas is at a high level, indicating that regional green and coordinated development have a strong competitive advantage. Among them, the sustainable development index of some regions is in the first echelon, including Guangdong, Shanghai, Zhejiang, Beijing and Jiangsu. Some regions, including Fujian, Tianjin, Hainan and Shandong, are in the second echelon of sustainable development and have strong development potential. In addition, the sustainable development level in the northeast region has a certain potential for improvement. And the sustainable development level of many provinces in the central region is not high and there is a large room for improvement. However, most provinces in the western region, including Qinghai, Gansu, Ningxia, Xinjiang and Guizhou, have a low level of sustainable development, and the overall green and coordinated development do not have a competitive advantage. On the whole, China's sustainable development presents obvious ladder-like characteristics and highlights the regional imbalance and inadequacy of regional green and coordinated development.

### Baseline regression analysis

To test the effect of public expenditure on sports on the region's sustainable development, this paper regresses Equation (1). Models 1 to 3 used the Tobit model, random effect model, and individual fixed effect model, respectively. Table 3 shows that the regression coefficient of public expenditure on sports to regional sustainable development is always positive, and it passes the confidence level test of more than 1%, indicating that increasing the level of public expenditure on sports can significantly promote regional sustainable development.

**Table 3:** Baseline regression result.

	**(1)**	**(2)**	**(3)**
	***sus***	***sus***	***sus***
*sportscl*	0.040***	0.037***	0.044***
	(0.007)	(0.007)	(0.007)
*energy*	−0.078***	−0.078***	−0.077***
	(0.008)	(0.007)	(0.008)
*patent*	0.013***	0.012***	0.015***
	(0.002)	(0.001)	(0.002)
*r&d*	0.001*	0.001	0.003***
	(0.001)	(0.001)	(0.001)
*gdp*	−0.175***	−0.187***	−0.157***
	(0.029)	(0.030)	(0.029)
*fdi*	0.029	0.041	0.009
	(0.031)	(0.031)	(0.033)
*upd*	0.031	0.018	0.045
	(0.030)	(0.029)	(0.031)
*constant*	0.023	0.067	−0.046
	(0.094)	(0.090)	(0.101)
σ_*u*_	0.055***		
	(0.008)		
σ_*e*_	0.027***		
	(0.001)		
Wald test	1328.38***	1277.11***	
adj. R^2^			0.785
*N*	360	360	360

The values in brackets are SDs. ***Indicates that the estimated coefficients are significant at the confidence level of 1%. **Indicates that the estimated coefficients are significant at the confidence level of 5%. *Indicates that the estimated coefficients are significant at the confidence level of 10%.

From the regression results of the control variables, the level of energy consumption has a significant negative impact on the sustainable development of the region, all of which passed the 1% significance test; that is, the reduction in the level of energy consumption per unit of GDP can promote the green, coordinated and sustainable development of the region. The regression coefficients of innovation climate on regional sustainable development are all positive, and all have passed the significance test with a confidence level of more than 1%, effectively verifying the importance of innovation environment construction to regional sustainable development. The regression coefficient of R&D support to the level of regional sustainable development is positive, and it passes the 1% confidence level test after controlling for individual effects, indicating that the emphasis on innovation investment will improve the level of regional sustainable development. The main reason for this result is that the increase in capital investment has promoted the agglomeration of talent and provided human capital for regional green innovation and coordinated industrial development. The regression coefficient of the economic growth rate on regional sustainable development is significantly negative, and it passes the 1% significance test, indicating that the extensive economic development model that simply pursued the growth rate in the past is incompatible with the sustainable development concept centered on green and coordination. Therefore, it is not advisable to blindly pursue the economic growth rate during the period of economic transformation, and more attention should be given to the sustainability of economic development. The regression coefficient of the level of openness to the outside world on the high-quality development of the region is positive. However, it has not passed the significance test, indicating that effectively attracting foreign investment is an essential measure for sustainable economic development, and attention should be given to the directional guidance of foreign investment. The regression coefficient of the degree of population agglomeration is positive, but it has not passed the significance test, indicating that while promoting population agglomeration, we should optimize the spatial structure of the regional population and build a benign relationship between urban population density and regional sustainable development.

To test the robustness of the effect of sports public expenditure on regional sustainable development, we conduct robustness tests by specifying the evaluation dimensions of sustainable development and replacing the measurement methods of core explanatory variables. The results are shown in Table 4. The explained variables of Model 1 and Model 2 are the specific subdivision dimension indicators of the sustainable development index, that is, the coordinated and green development index. The regression coefficient of the public expenditure on sports to the level of regional innovation development and the degree of coordinated development is significantly positive at the 1% confidence level, indicating that the public expenditure on sports has a comprehensive role in promoting the sustainable development of the region. Specifically, the influence coefficient of sports development on coordinated development is relatively large (0.072). On the one hand, it shows that the government has strengthened the construction of urban and rural sports infrastructure through sports public expenditure and improved the level of coordinated development among regions. On the other hand, the government guides the development of the sports economy through public financial expenditure and promotes the continuous improvement of the coordinated development level of regional industries. The impact of sports development on green development is also significant, indicating that while promoting the development of green sports, the government also promotes the level of regional green innovation and promotes the development of the regional eco-friendly green economy. To alleviate the problem of autocorrelation of residuals among individuals, Model 3 performs cluster regression on the standard errors of coefficients at the provincial level. The results show that the regression coefficient of sports public expenditure on regional sustainable development is positive at the 5% significance level. In addition, we use the fixed asset investment in sports to replace the regional sports public financial expenditure and measure the level of government sports public expenditure by calculating the stock and taking the logarithm. Model 4 shows that the positive effect of sports development on regional sustainable development is still significant at the 1% confidence level.

**Table 4:** The robustness checks.

	**(1)**	**(2)**	**(3)**	**(4)**
	***coordination***	***greeness***	***sus***	***sus***
*sportscl*	0.072***	0.037***	0.044**	
	(0.012)	(0.009)	(0.018)	
*sportscl1*				0.035***
				(0.003)
*constant*	−0.353**	0.045	−0.046	0.009
	(0.164)	(0.117)	(0.247)	(0.048)
*control*	YES	YES	YES	YES
Province fixed effect	YES	YES	YES	YES
adj. *R*^2^	0.581	0.747	0.803	0.832
*N*	360	360	360	360

The values in brackets are SDs. ***Indicates that the estimated coefficients are significant at the confidence level of 1%. **Indicates that the estimated coefficients are significant at the confidence level of 5%. *Indicates that the estimated coefficients are significant at the confidence level of 10%.

The paper eliminates the endogeneity problem through the tool variable method and selects the tool variable by using the lagged one-period public expenditure on sports (*L.sportscl*). Specifically, we use the IV-two-stage least squares regression (IV-2SLS) method, optimal generalized methods of moments (GMM) and the iterative GMM to alleviate the potential endogeneity issue. The specific analysis results are shown in Table 5. *Shea's adj.partial R*^2^ value is 0.9822, the F test statistic is 18485.1, the *p*-value is 0.000, and the minimum eigenvalue statistic 18071.9 is much larger than 10, indicating that there is no problem with weak instrumental variables. From the estimation results of the two-stage least squares method (IV-2SLS), the optimal GMM method and the iterative GMM method from Models 1 to 3, it can be seen that the regression coefficient of public expenditure on sports for regional sustainable development is significantly positive at the 1% confidence level. The results of the optimal GMM estimation and iterative GMM estimation are basically consistent with the estimation results of IV-2SLS, and the increased emphasis on sports development can significantly improve the level of regional sustainable development. *Hypothesis 1* is verified.

**Table 5:** Endogeneity test.

	**(1)**	**(2)**	**(3)**
	***sus***	***sus***	***sus***
*sportscl*	0.014***	0.014***	0.014***
	(0.005)	(0.005)	(0.005)
*constant*	0.416***	0.416***	0.416***
	(0.064)	(0.064)	(0.064)
*Shea's adj. partial R2*	0.9822		
*Robust F*	18485.1		
	[0.000]		
Minimum eigenvalue statistic	18071.9		
adj. R^2^	0.736	0.736	0.736
*N*	330	330	330

The values in brackets “()” are SDs. The values in brackets “[]” are the value of p of the corresponding test statistics. The values in brackets are SDs. ***Indicates that the estimated coefficients are significant at the confidence level of 1%. **Indicates that the estimated coefficients are significant at the confidence level of 5%. *Indicates that the estimated coefficients are significant at the confidence level of 10%.

### Threshold effect test of regional development level

To further explore the impact of government sports public expenditure on regional sustainable development under different economic development levels and to examine the threshold effect of regional economic development levels, we draw on the threshold model constructed by Hansen (43) and use regional per capita GDP as the threshold variable to conduct regression analysis. From the results of the threshold effect self-sampling test and the threshold value estimation in Table 6, it can be seen that there is a double threshold effect in the impact of the regional economic development level on the sustainable development of the region. Table 7 shows the regression results of the impact of government sports public expenditure on regional sustainable development under different economic development levels. When per capita GDP is < 20,323, the regression coefficient of public expenditure on sports to regional sustainable development is 0.025. When the per capita GDP is in the range of 2.0323 to 41,206, the regression coefficient of public expenditure on sports to the region's sustainable development is 0.028. When per capita GDP is higher than 41,206, the regression coefficient of public expenditure on sports to regional sustainable development is 0.030. On the whole, with the continuous improvement of the regional development level, the regression coefficient of the public expenditure on sports to the regional sustainable development index has continued to increase, and the significance has continued to increase. This shows that in the process of improving the income level of regional residents, the impact of the sports economy on regional green and coordinated development has gradually deepened, and the public expenditure on sports has a nonlinear impact on regional sustainable development. *Hypothesis 2* is verified.

**Table 6:** The existence test of the threshold effect of the regional development level.

**Threshold variables**	**Threshold type**	**F value**	**P value**	**Threshold value**	**BS times**
Level of regional development	Single threshold	33.34	0.0367	1.3890	300
	Double threshold	40.03	0.0033	2.0323	
	Triple threshold	29.11	0.3800	4.1206	

**Table 7:** Threshold regression results.

	**(Double threshold)**
	***sus***
*sportscl* (*gdp_per ≤* 2.0323)	0.025*
	(0.015)
*sportscl* (2.0323 * < gdp_per ≤* 4.1206)	0.028*
	(0.015)
*sportscl* (*gdp_per >* 4.1206)	0.030**
	(0.014)
*constant*	0.138
	(0.198)
control	YES
adj. R^2^	0.838
N	360

The values in brackets are SDs.

^***^Indicates that the estimated coefficients are significant at the confidence level of 1%.

^**^Indicates that the estimated coefficients are significant at the confidence level of 5%.

^*^Indicates that the estimated coefficients are significant at the confidence level of 10%.

### Analysis of the moderating effect of the level of finance development

In the environment where the market plays a decisive role in the allocation of economic resources, to verify whether the effect of local government sports public expenditure on regional sustainable development is affected by the level of market financial development, according to Equation (2) and Equation (3), we introduce the interaction term between the level of financial development and public expenditure on sports for regression analysis. The results are shown in Table 9. Model 1 and Model 2 test the influence of the market financial development level on sustainable development and the moderating effect on the promotion effect of sports public expenditure increase, respectively. The results show that the regression coefficients of the market finance development level, the interaction term of the market finance development level and sports public expenditure are all significantly positive and have passed the 1% confidence level test. This shows that the market financial power can effectively promote the region's sustainable development, and the improvement of the development level of the market finance enhances the promotion of the public expenditure on sports to the sustainable development of the region. To further verify the strengthening effect of market financial development, we decompose the sustainable development index and replace the sustainable development index with the regional green development and coordinated development index. The regression results are model 3 and model 4. The regression coefficients of the interaction term between the market finance development level and sports public expenditure on regional green development and coordinated development are all significantly positive at the 1% confidence level, indicating that the strengthening effect of the market finance development level on the promotion effect of sports public expenditure is robust. *Hypothesis 3* is verified.

### Analysis of the transmission mechanism

It can be seen from the above analysis that public expenditure on sports has a significant role in promoting the green, coordinated and sustainable development of the region. Whether the government's guidance on the development of the sports economy can activate the consumption of residents, effectively build an internal circulation market, and promote the region's sustainable development needs further verification. We adopt the step-by-step analysis of the mediating effect to regress Equation (4) and Equation (5), and the results are shown in Table 8. From the regression results of the mediating effect of residents' consumption level, it can be seen that the regression coefficient of the public expenditure of sports undertakings on residents' consumption level is significantly positive at the 1% confidence level. This result means that the government activates regional residents' consumption by increasing public expenditure on sports, thereby promoting regional sustainable development. Its mediating effect is 0.022 (0.345 × 0.064). Specifically, the regression coefficients of regional residents' consumption on the coordinated development and green development index are all significantly positive at the 1% confidence level. The mediating effects were 0.038 (0.345 × 0.109) and 0.017 (0.345 × 0.051), respectively. The results show that public expenditure on sports can improve regional development's green and coordination levels by activating the level of regional consumption. The enhancement of coordination stems from the construction of public sports infrastructure. By increasing public expenditure on sports, the government has provided a soft environment guarantee for residents' sports and healthy activities and alleviated the pressure of basic life and work and has narrowed the gap in regional development through transfer payments and promoted coordinated regional development. The concept of green sports is rooted in the development of sports. The green development orientation of the sports industry itself has enhanced the vitality of regional green innovation, increased the publicity of green and low-carbon development of the regional economy, created an atmosphere of green development, and then promoted green development. It can be seen from the results of the mediating effect test that the results of the Sobel test and the bootstrap test are consistent. Moreover, in the mechanism test of regional sustainable development, coordinated development and green development, the mediating effect of regional residents' consumption is established at the 1% confidence level. *Hypothesis 4* has been verified; that is, the increase in government public expenditure on sports can improve the level of regional consumption, activate the internal circulation market, and promote the sustainable development of the region.

**Table 8:** The regression result of the mediating effect based on residents' consumption levels.

	**(1)**	**(2)**	**(3)**	**(4)**
	***consump***	***sus***	***coordination***	***greeness***
*sportscl*	0.345***	0.022***	0.034***	0.019**
	(0.038)	(0.008)	(0.013)	(0.009)
*consump*		0.064***	0.109***	0.051***
		(0.010)	(0.016)	(0.012)
*constant*	−3.217***	0.161	−0.004	0.208*
	(0.521)	(0.101)	(0.163)	(0.120)
*control*	YES	YES	YES	YES
Province fixed effect	YES	YES	YES	YES
adj. R^2^	0.857	0.808	0.630	0.760
N	360	360	360	360
Sobel test		0.022***	0.038***	0.017***
		(0.004)	(0.007)	(0.005)
Bootstrap test		0.022***	0.038***	0.017***
		(0.005)	(0.008)	(0.005)

The values in brackets are SDs. ^***^Indicates that the estimated coefficients are significant at the confidence level of 1%. ^**^Indicates that the estimated coefficients are significant at the confidence level of 5%. ^*^Indicates that the estimated coefficients are significant at the confidence level of 10%.

**Table 9:** The regression result of the moderating effect based on the level of development of finance.

	**(1)**	**(2)**	**(3)**	**(4)**
	***sus***	***sus***	***Coordination***	***Greenness***
*sportscl*	0.035***	0.033***	0.046***	0.031***
	(0.008)	(0.008)	(0.013)	(0.009)
*fin*	0.592***			
	(0.171)			
*fin × sportscl*		0.047***	0.107***	0.026*
		(0.013)	(0.021)	(0.015)
*constant*	0.038	0.059	−0.113	0.104
	(0.103)	(0.104)	(0.165)	(0.121)
*control*	YES	YES	YES	YES
Province fixed effect	YES	YES	YES	YES
adj. R^2^	0.792	0.792	0.612	0.749
N	360	360	360	360

The values in brackets are SDs. ^***^Indicates that the estimated coefficients are significant at the confidence level of 1%. ^**^Indicates that the estimated coefficients are significant at the confidence level of 5%. ^*^Indicates that the estimated coefficients are significant at the confidence level of 10%.

## Conclusions and policy implications

Sustainable development is the development with the goal of improving the level of green and coordinated development in the region, and the construction of sports at a new stage of development is an important driving force for sustainable development in the region. This paper constructs a regional sustainable development index according to the green coordinated development goal and theoretically analyses and empirically tests the impact of government sports public expenditure on regional sustainable development. The study found the following: (1) China's sustainable development presents obvious ladder-like characteristics and highlights the regional imbalance and inadequacy of regional green and coordinated development. (2) The government's increase in public expenditure on sports can effectively promote the sustainable development of the region and will significantly enhance the level of green and coordinated development in the region. (3) The public expenditure on sports has a non-linear impact on the region's sustainable development. With the continuous improvement of the regional economic development level, the government's public expenditure on sports has an increasing role in promoting the region's sustainable development. (4) The improvement of the development level of market finance can strengthen the effect of the government's public expenditure on sports on the sustainable development of the region. (5) The government's increase in public expenditure on sports has significantly promoted the greenness and coordination of regional development by stimulating regional residents' consumption and can achieve sustainable regional development.

Based on the research conclusions and the goal of formulating a more perfect sports development plan in the post-COVID era, this paper has the following policy implications. First, the government should fully explore the vast market potential and economic value of sports, give continuous fiscal support to sports, formulate sports infrastructure construction plans that are suitable for the stage of economic development, and improve the level of sustainable development of the region. On the one hand, the government should actively promote the green concept in the growth and development of sports and use green sports to activate the vitality of regional green innovation, enhance regional awareness of ecological environmental protection, and reduce energy consumption, thereby promoting regional green development. On the other hand, in the period of regional economic transformation and industrial structure adjustment, the government should guide social capital to enter the sports and health industry through fiscal support, promote the vigorous development of the green service industry in the form of industrial support, and rationally optimize the regional industrial structure. In addition, the local government should narrow the gap in regional economic development through sports finance transfer payments and then promote regional coordinated development. Second, we should fully realize that with the improvement of the regional economic development level, the continuous fiscal support for sports can more effectively promote regional sustainable development. At the same time, we should find synergy between market financial forces and government fiscal support, amplify the positive externalities of government investment in sports, and achieve sustainable regional development. Third, we should pay attention to the mediating effect of residents' consumption on regional sustainable development. Public expenditure on sports should be expanded, the transformation of residents' consumption quality and consumption structure from the supply side should be guided, the deep integration of the regional sports service industry and economic development should be promoted, and the level of regional sustainable development should be improved.

## Data availability statement

The original contributions presented in the study are included in the article/supplementary material, further inquiries can be directed to the corresponding author/s.

## Author contributions

The study was designed by DW in collaboration with all co-authors. The writing and editing work of the corresponding parts and the major revisions of this article were completed by DW and XH. EZ developed the conceptualization and research methodology and analyzed the results. PQ criticized the defects of the draft, reviewed the relevant literature, supervised the work, and acquired funds for the research. All authors contributed to the article and approved the submitted version.

## Funding

This research was supported by the National Social Science Funding of China (Project Name: Research on the Mutual Symbiosis Mechanism between the Development of Sports in Northeast China and the Revitalization of New Northeast China; Grant Number: 21BTY082).

## Conflict of interest

The authors declare that the research was conducted in the absence of any commercial or financial relationships that could be construed as a potential conflict of interest.

## Publisher's note

All claims expressed in this article are solely those of the authors and do not necessarily represent those of their affiliated organizations, or those of the publisher, the editors and the reviewers. Any product that may be evaluated in this article, or claim that may be made by its manufacturer, is not guaranteed or endorsed by the publisher.

## Footnotes

^1^ https://theory.gmw.cn/2020-11/10/content_34354489.htm

^2^ https://www.cnipa.gov.cn/col/col1510/index.html

## References

[1] Wang F Lu Y Li J Ni J. Evaluating environmentally sustainable development based on the PSR framework and variable weigh analytic hierarchy process. Int J Environ Res Public Health. (2021) 18:2836. 10.3390/ijerph18062836 33802188 PMC8001335

[2] Cai C Qiu R Tu Y. Pulling off stable economic system adhering carbon emissions, urban development and sustainable development values. Front Public Health. (2022) 10:814656. 10.3389/fpubh.2022.814656 35223738 PMC8866235

[3] Martin K Mullan Z Horton R. Human health and environmental sustainability: the 21st century's grand challenges. Lancet Global Health. (2016) 4 (Suppl. 1): S1–2. 10.1016/S2214-109X(16)30001-8 27068196

[4] Cumming GS Cramon-Taubadel S. Linking economic growth pathways and environmental sustainability by understanding development as alternate social–ecological regimes. Proc Nat Acad Sci. (2018) 115:9533–8. 10.1073/pnas.1807026115 30185564 PMC6156676

[5] Xu XC Lei ZK Dou YY Liu SC. Research on gap of balanced development between the North and the South of China—analysis based on “China Balanced Development Index”. China Industrial Economics. (2021) (02):5–22. 10.19581/j.cnki.ciejournal.2021.02.011

[6] Yu X Liang Z Fan J Zhang J Luo Y Zhu X. Spatial decomposition of city-level CO2 emission changes in beijing-tianjin-hebei. J Clean Prod. (2021) 296:126613. 10.1016/j.jclepro.2021.126613

[7] Yang K. The construction of sports culture industry growth forecast model based on big data. Personal Ubiquitous Computing. (2020) 24:5–17. 10.1007/s00779-019-01242-z

[8] Lu C Ren W Jiang L Xue B. Modelling impact of climate change and air pollution in cities. Proc Inst Civ Eng: Eng Sus. (2017) 170:133–140. 10.1680/jensu.16.00002

[9] Ding L Shao Z Zhang H Xu C Wu D. A comprehensive evaluation of urban sustainable development in China based on the TOPSIS-entropy method. Sustainability. (2016) 8:746. 10.3390/su808074630658429

[10] Folke C. Resilience: the emergence of a perspective for social-ecological systems analyses. Global Environ Change. (2006) 16:253–67. 10.1016/j.gloenvcha.2006.04.002

[11] Singh GK Siahpush M. Widening rural–urban disparities in all-cause mortality and mortality from major causes of death in the Usa, 1969-2009. J Urban Health. (2014) 91:272–92. 10.1007/s11524-013-9847-2 24366854 PMC3978153

[12] Ameer W Amin A Xu H. Does institutional quality, natural resources, globalization, and renewable energy contribute to environmental pollution in China? Role of financialization. Front Public Health. (2020) 10:849946. 10.3389/fpubh.2022.849946 35433588 PMC9008753

[13] Ucak H Aslan A Yucel F Turgut A. A dynamic analysis of CO2 emissions and the gdp relationship: empirical evidence from high-income oecd countries. Energy Sources Part B Econo Plann Policy. (2015) 10:38–50. 10.1080/15567249.2010.514586

[14] Ahmad M Akram W Ikram M Shah AA Rehman A Chandio AA. Estimating dynamic interactive linkages among urban agglomeration, economic performance, carbon emissions, and health expenditures across developmental disparities. Sustain Product Consumption. (2020) 26:239–55. 10.1016/j.spc.2020.10.006

[15] Lin B Zhu J. Energy and carbon intensity in China during the urbanization and industrialization process: a panel var approach. J Clean Prod. (2017) 168:780–90. 10.1016/j.jclepro.2017.09.013

[16] Bekhet HA Latif NWA. The impact of technological innovation and governance institution quality on Malaysia's sustainable growth: evidence from a dynamic relationship. Technol Soc. (2018) 54:27–40. 10.1016/j.techsoc.2018.01.014

[17] Feng W Yu K. Government governance, legal environment and sustainable economic development. Sustainability. (2014) 6:2248–63. 10.3390/su6042248

[18] Tang D Li ZJ Bethel BJ. Relevance analysis of sustainable development of China's Yangtze river economic belt based on spatial structure. Int J Environ Res Public Health. (2019) 16:3076. 10.3390/ijerph16173076 31450878 PMC6747499

[19] Gu W Wang J Hua X Liu Z. Entrepreneurship and high-quality economic development: based on the triple bottom line of sustainable development. Int Entrepreneur Manag J. (2021) 17:1–27. 10.1007/s11365-020-00684-9

[20] Dabbous A Tarhini A. Does sharing economy promote sustainable economic development and energy efficiency? Evidence from OECD countries. J Innovation Knowledge. (2021) 6:58–68. 10.1016/j.jik.2020.11.001

[21] Hall JC Humphreys BR Pyun H. An inventory of sports economics courses in the us. Int J Sport Finance. (2017) 12:3–13. Available online at: http://busecon.wvu.edu/phd_economics/pdf/15-49.pdf

[22] Santos JMS García PC. A bibliometric analysis of sports economics research. Int J Sport Finance. (2011) 6:222–44. Available online at: https://www.proquest.com/scholarly-journals/bibliometric-analysis-sports-economics-research/docview/889138877/se-2

[23] Gu D Wu Y Dai Z. A study of the changes in government functions in elite sport development in China, 1949-2012. Int J History Sport. (2015) 32:1353–13. 10.1080/09523367.2015.1066993

[24] Jung M Jung H Hong N Kim Y. The effect of government supports in marine industry and sport club membership on the networking behavior and participatory activities. J Sport Leisure Stud. (2010) 42:397–407. 10.51979/KSSLS.2010.11.42.397

[25] Mao LL Huang H. Social impact of formula One Chinese Grand Prix: a comparison of local residents' perceptions based on the intrinsic dimension. Sport Manag Review. (2016) 19:306–18. 10.1016/j.smr.2015.08.007

[26] Zhao Y. Sports enterprise marketing and financial risk management based on decision tree and data mining. J Healthc Eng. (2021) 8:7632110. 10.1155/2021/7632110 34691380 PMC8536418

[27] Liu JY. Analysis of the synergistic impacts of public services expenditure on economic growth and social equity. 2012 International Conference on Management Science and Engineering 19th Annual Conference Proceedings. (2012). p. 1904–10. 10.1109/ICMSE.2012.6414431

[28] Dallmeyer S Wicker P Breuer C. The relationship between sport-related government spending and sport and exercise participation: the role of funding size, period, and consistency. Int J Health Promotion Edu. (2018) 56:237–47. 10.1080/14635240.2018.1452623

[29] Kim S. Assessing economic and fiscal impacts of sports complex in a small us county. Tourism Economics. (2021) 27:455–65. 10.1177/1354816619897151

[30] Lee YH Watanabe N. Sports economics and management of Asian sports business. J Global Sport Manag. (2019) 4:121–7. 10.1080/24704067.2018.155302329961442

[31] Hee H Eob Y Choi S. An analysis on the application of the fourth industrial revolution and the sports convergence industry. Korean J Sport. (2020) 18:821–34. 10.46669/kss.2020.18.3.075

[32] Prest AR. [Review of the theory of public finance. by R A Musgrave]. Economic J. (1959) 69:766–70. 10.2307/2227672

[33] Rothbarth E. [Review of the conditions of economic progress. by C Clark]. Economic Jo. (1941) 51:120–4. 10.2307/2225658

[34] Zhang BH Li JF Li GL Yang NS Chen HM Li ZK. The research on the status and role of China's sports industry in the national economy. China Sport Sci. (2007) 27:22–30. 10.16469/j.css.2007.04.003

[35] Meek A. An estimate of the size and supported economic activity of the sports industry in the United States. Sport Marketing Quarter. (1997) 16:23–32. Available online at: https://xueshu.lanfanshu.cn/scholar?q=related:d_Y9fQNZkgwJ:xueshu.lanfanshu.cn/&scioq=An+estimate+of+the+size+and+supported+economic+activity+of+the+sports+industry+in+the+United+States.&hl=zh-CN&as_sdt=0,5

[36] Watson WG. Western economies in transition: Structural change and adjustment policies in industrial countries, (editors). Hudson Institute Studies on the Prospects for Mankind. Boulder, Colorado: Westview Press. (2015) p. 218–21.

[37] Bolcárová P Kološta S. Assessment of sustainable development in the EU 27 using aggregated SD index. Ecological Indicators. (2015) 48:699–705. 10.1016/j.ecolind.2014.09.001

[38] Wang WC Jiao YJ. Spatial evolution characteristics and the spatial spillover effect of sports public fiscal expenditure in China. J Beijing Sport Univer. (2020) 43:74–83. 10.19582/j.cnki.11-3785/g8.2020.06.008

[39] Borio CE Lombardi MJ Zampolli F. Fiscal Sustainability the Financial Cycle. (2016). Available at online at: https://ssrn.com/abstract=2752560 (accessed September 9, 2022).

[40] Modigliani F Cao SL. The Chinese saving puzzle and the life-cycle hypothesis. J Econ Lit. (2004) 42:145–70. 10.1257/002205104773558074

[41] Chao CC Laffargue JP Yu E. The Chinese saving puzzle and the life-cycle hypothesis: a revaluation. China Economic Rev. (2011) 22:108–20. 10.1016/j.chieco.2010.09.004

[42] Baron RM Kenny DA. The moderator-mediator variable distinction in social psychological research: conceptual. Strategic and statistical considerations. J Pers Soc Psycho. (1986) 51:1173–82. 10.1037/0022-3514.51.6.1173 3806354

[43] Hansen BE. Threshold effects in non-dynamic panels: estimation, testing, and inference. J Econom. (1999) 93:345–68. 10.1016/S0304-4076(99)00025-1

